# Bridging the AI Divide in Drug Discovery: Practical Lessons from Capacity Strengthening in Africa

**DOI:** 10.1021/acs.jmedchem.6c01429

**Published:** 2026-08-07

**Authors:** Jason Hlozek, Miquel Duran-Frigola, Nicola Elliott-Wong, Rudolf Stander, Susan Winks, Gemma Turon, Kelly Chibale

**Affiliations:** † Holistic Drug Discovery and Development (H3D) Centre, 37716 University of Cape Town, Cape Town, Western Cape 7700, South Africa; ‡ 717261 Ersilia Open Source Initiative, Barcelona 08039, Spain; § H3D Foundation, Cape Town, Western Cape 7700, South Africa; ∥ South African Medical Research Council Drug Discovery and Development Research Unit, Department of Chemistry & Institute of Infectious Disease and Molecular Medicine, University of Cape Town, Cape Town, Western Cape 7925, South Africa

## Abstract

Artificial Intelligence (AI) has become an integral part of drug discovery in high-income countries. However, these advances have yet to be broadly adopted by research ecosystems in many low- and middle-income countries in Africa, where understudied infectious diseases, compounded by a growing noncommunicable disease burden, cause millions of deaths each year. Given adequate support and appropriate models AI could play a transformative role in under-resourced biomedical research ecosystems in Africa, democratizing drug discovery research. There is an urgent need to invest in building the critical infrastructure, tools, and capacity required. To drive this agenda forward, Ersilia, the H3D Centre, and H3D Foundation with support from the AI2050 program at Schmidt Sciences hosted a series of in-person and online workshops in Africa to grow the AI skills base for medicinal chemistry and enable adoption across regional research hubs. In this perspective article, we discuss the lessons learnt from this program, the fit-for-purpose open-source tools developed, and key considerations for future initiatives.

## Graphical abstract

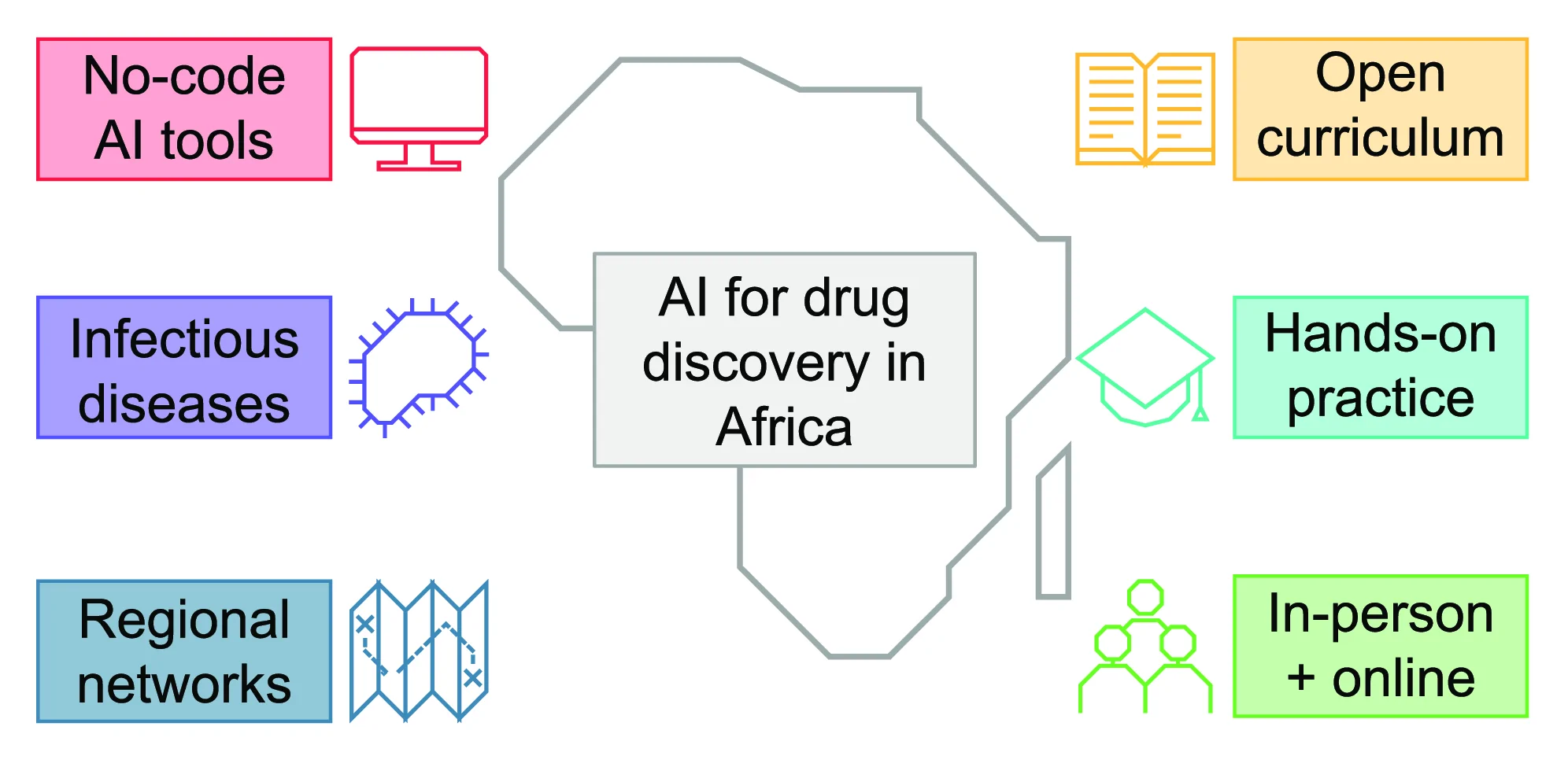

## Significance, Impact, and Innovation Statement

This Perspective article addresses the inequities in access to artificial intelligence (AI) for drug discovery in Africa and demonstrates how locally embedded, open-source AI programs can meaningfully bridge this gap. The article, drawing from a two-year program targeting African scientists, outlines how capacity strengthening efforts for AI tools can enable researchers in resource-limited settings to accelerate drug discovery for endemic diseases and enable equitable AI adoption by regional science hubs on the African continent.

## Introduction

Artificial Intelligence (AI) and Machine Learning (ML) is transforming biomedical research.^1^ Indeed, computational methods have long been used to sift through the chemical space in search of novel bioactive compounds, and the wave of AI advancements is increasing capabilities exponentially. Recent technological and architectural breakthroughs, which have enabled the invention of models like AlphaFold^2^ or Boltz,^3^ have consolidated the adoption of AI methods across the innovative pharmaceutical industry, biotech start-ups and academia in the Global North. However, a combination of limited infrastructure, expertise and accessible data availability has hampered commensurate AI adoption in underserved scientific communities,^4^ where, paradoxically, AI/ML could deliver the greatest value in reducing the time and cost of drug discovery.^5^ It is therefore essential that the scientific community comes together to bridge this gap before the inequalities in Global Health research further deepen. Concerted AI/ML training initiatives in Africa have been established for bioinformatics^6^ and, more recently, in structural biology,^7^ however, similar training for small-molecule drug discovery remains limited. Acknowledging this need, the University of Cape Town Holistic Drug Discovery and Development (H3D) Centre in South Africa, the first, largest and only integrated drug discovery organization on the continent (https://h3d.uct.ac.za), its associated nonprofit, the H3D Foundation (https://h3dfoundation.org), and the Ersilia Open Source Initiative (https://ersilia.io), a Spanish nonprofit organization fueling research in infectious diseases through AI, joined forces in 2023 to build a collaborative framework for AI/ML capacity building across Africa. This work is embedded within the AI2050 program at Schmidt Sciences, a program that supports researchers addressing ambitious research problems aiming to ensure AI benefits society by 2050, of which Prof. Kelly Chibale, Director of the H3D Centre and CEO of H3D Foundation, is a Senior Fellow.

Drug discovery is a complex process that requires collaboration of experts across many scientific domains.^8^ However, open source AI/ML tools are often developed by research groups that are isolated from the formal drug discovery process, creating a disconnect between the tool developers and its end users. Furthermore, the use of these tools typically requires computational expertise and knowledge of ML concepts which drug discovery researchers often lack, limiting their ability to effectively implement AI/ML methods in their projects. Therefore, while AI/ML is well-suited to analyzing the data sets produced in drug discovery research, its adoption remains limited.^9^ To address this gap, we designed a training program to enable lab-based scientists in Africa to develop the necessary skills for greater AI/ML adoption. Our goal is to deliver core AI/ML concepts and demonstrate the applicability of no-code tools to bridge the gap between wet- and dry-lab research and ultimately accelerate drug discovery pipelines.

Following an introductory course conducted in early 2024 in Zambia during the H3D Symposium, we crafted a scalable, reproducible and open curriculum designed as a four-day workshop, which we implemented on-site across different geographical regions (West Africa in 2024 and East Africa in 2025) as well as online (in 2025) to facilitate broader participation. In total, the program has reached more than 300 participants from 22 African countries. Below, we present our perspective on the critical elements of effective AI/ML training and how these can be translated into sustained adoption in research projects.

## Enabling AI Adoption in Africa

### Target Audience and Hosts

The training framework was built on three pillars: infectious diseases, small molecule drug discovery and AI/ML. Narrowing the scope to a defined domain of applicability was intentional, allowing us to leverage our research expertise and to deliver targeted training using relevant real-world examples with the purpose of promoting the greatest impact for the workshop participants.

The target audience included medicinal chemists, biologists, pharmacists and computational biologists. All courses received hundreds of applications from scientists at all career stages (PhD student to principal investigator, R1 to R4 in the European Framework for research careers), which demonstrates an appetite for this kind of training. For in-person workshops, candidate selection was primarily based on the relevance of the course content to the applicant’s ongoing research, assessed by their motivation letters and previous research projects. Gender, career stage and geographic location were also factors we considered to select representative and diverse cohorts. Participants were intentionally drawn from three tiers: host city (15), broader host country (10) and neighboring countries (5). This structure supported local capacity strengthening as well as regional network building, and reduced travel costs (Figure 1A). To ensure egalitarian participation, the program was fully sponsored, including the registration fee, flight tickets, accommodation and meals.

**Figure 1:**
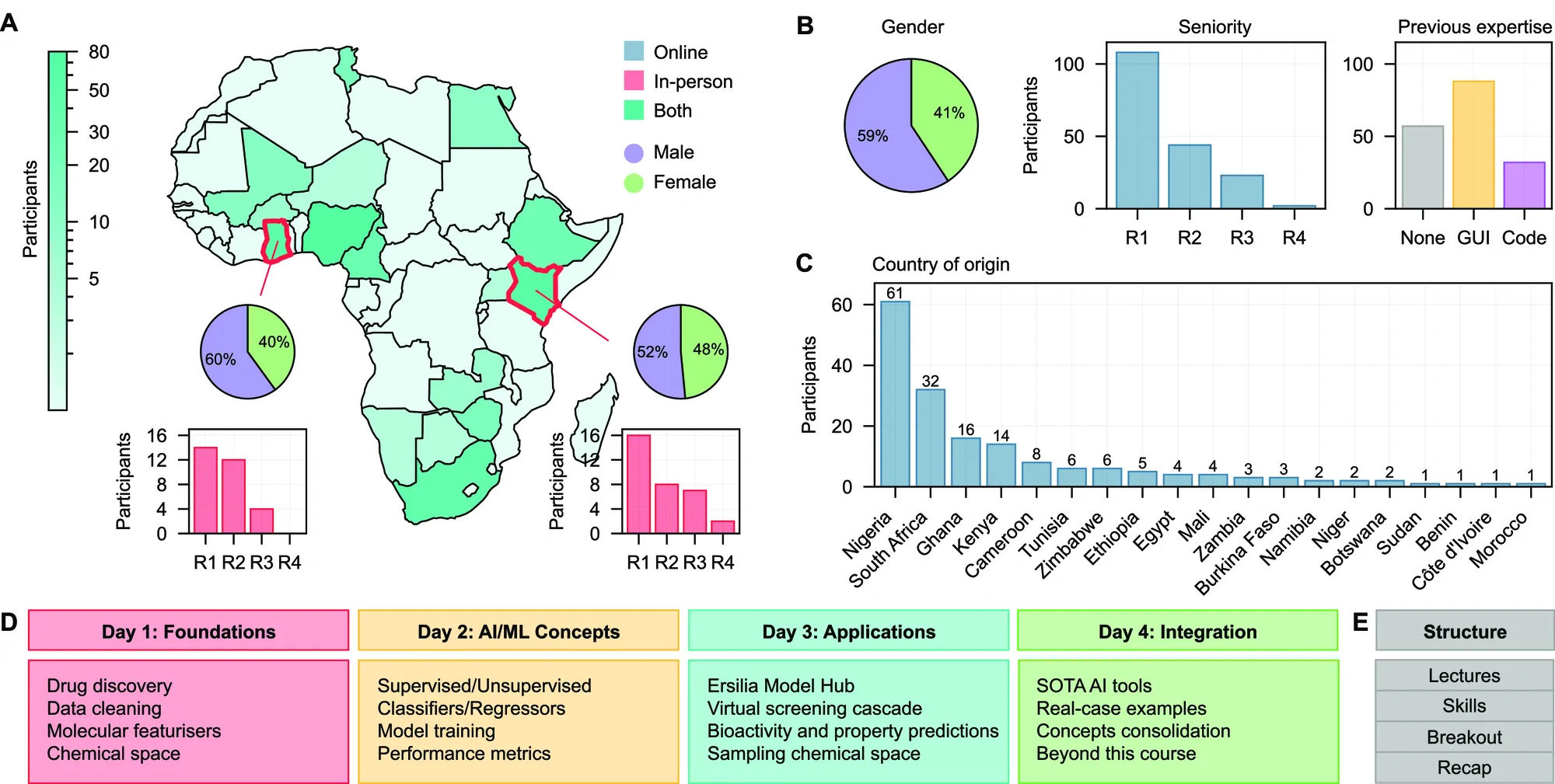
Capacity building activities in Africa. (A) Number of participants across the African continent by country. The red insets highlight Ghana and Kenya, where two in-person workshops took place. The pie charts indicate gender proportion of each in-person workshop cohort. The corresponding bar plots categorize participants by seniority (R1: first-stage, R2: recognized, R3: established, R4: leading). (B) Demographics of the online workshop, including gender proportion, seniority, and level of AI exposure prior to the meeting. (C) Country-of-origin ranked by number of participants in the online workshop. (D) General scheme of the workshop content across the 4-days. (E) Typical day structure.

In addition, we partnered with local host institutions who could provide logistical support (such as on-the-ground transport and letters of invitation for visa applications), extended networking and communication and support to align the program with the local scientific context. Among other tasks, the local hosts nominated guest lecturers that were invited to deliver keynote talks on drug discovery topics that provided context for the AI topics each day. This not only broadened the program’s reach but also ensured that the training was meaningfully embedded within the local scientific milieu. The guest lecturers were key people in local organizations and in several instances they chose to stay on as participants for the full workshop, building rapport between participants and facilitators and directly growing institutional capacity for AI/ML tools. Finally, networking was embedded throughout the program via structured breaks and dedicated events, which participants identified as valuable for building collaborations.

Given the high volume of applications to each in-person workshop combined with the limitations of the in-person workshop capacity (only 10–20% of applicants could be accommodated), we decided to adapt our workshop to an online webinar format to reach a broader audience. Application to the online workshop remained free of charge and unrestricted, reaching over 160 researchers who, in most cases, had no, or limited, computational expertise (Figure 1B). Participation in the online course was Africa-wide, with an over-representation of Nigerian and South African attendees (Figure 1C). Webinars were recorded and hosted online, ensuring poor Internet connectivity was not a barrier to access and enabling participants to review the material as required. While the webinars did not cover the full course content, they offered the core components combined with a daily hands-on activity. The webinar recordings are hosted on the H3D Foundation YouTube channel with the course materials freely available on the Ersilia platform.

### Course Design Considerations

The course structure was designed around three core objectives: (i) introducing fundamental AI/ML concepts and its challenges when applied to drug discovery, (ii) enabling the use of open-source ready-to-use AI/ML tools, and (iii) fostering interdisciplinary collaboration. The final structure of the program was shaped by the lessons learned from prior training efforts to lower the barrier for AI/ML adoption.

#### Lowering Technical Barriers

Lab-based scientists typically do not have concomitant programming expertise or familiarity with non-Windows operating systems. Therefore, even low-code tools, such as the Google Colab Notebooks used in our earlier training workshops (Event Fund by Code for Science and Society, Cape Town, 2022), presented a significant barrier to adoption. We have since shifted toward developing graphical user interfaces (GUIs), allowing participants to interact with AI/ML models without requiring coding expertise, while still engaging critically with outputs.

#### Integrating Drug Discovery Knowledge

While the focus of the training is on AI/ML for chemical data sets, broader drug discovery context is required for understanding why these approaches are useful and when they could be applied in a drug discovery project. Drug discovery is a broad topic, and the field is still in a nascent stage of development in Africa. Prior familiarity with drug discovery and infectious disease concepts could not be assumed, therefore, we realized that we would need to teach the AI/ML material in parallel to the drug discovery context in which it could be applied.

#### Iterative, Hands-On Learning

Course materials were a mix of lecture-style content and hands-on practical exercises. Importantly, we found that setting a small deliverable for the end of a practical task kept groups focused. Allowing groups to do the task independently with minimal input from facilitators until the subsequent consolidation session was an effective formula to promote learning and engagement. Examples were best received when they were as close to real-world scenarios as possible.

#### Pace of Content Delivery

Delivery of course content was designed to be adaptable depending on the progress of the cohort. We deemed it preferable to establish selected core concepts instead of attempting to teach many concepts and risking participants being overwhelmed with too many new concepts.

#### Facilitated Interaction and Networking

Facilitators were available for informal chats and Q&A during the entire course, and, in addition, a welcome networking activity and a networking dinner were arranged to encourage discussion, reflection and connection. While more challenging in online settings, we fostered interaction though encouraging participants to post questions in the chat which were later shared with the corresponding answers in a document drawn up by the facilitators.

#### Certification and Motivation

Offering a certificate of participation to participants who completed 80% or more of the course boosted the participant’s ownership of what they had learnt while also amplifying awareness through encouraging social media activity as participants proudly posted photos of their certificates.

#### Operational Support

Dedicated logistical support (including through registration, on-site during the course, as well as in collecting participant feedback) enabled smooth program delivery and allowed facilitators and participants to focus on course content.

### Training Materials and Course Structure

Our curriculum was divided into four sections and the content of each day was designed to build on the previous day’s learning. The course structure could be understood from the medicinal chemist perspective (introduction to drug discovery, the chemical space, bioactivity prediction and generation of new molecules) as well as the computational researcher (data collection and curation, introduction to supervised and unsupervised ML and generative AI methods) (Figure 1D). The need to teach drug discovery concepts as context for the AI/ML content of each day presented an opportunity to leverage the knowledge and reputation of local experts who, each morning, delivered presentations on their area of expertise. To foster meaningful learning and collaboration, each day included a tailored combination of activities for the content delivery of each section: classroom-style lectures to introduce the concepts, a skill-based session where course facilitators guided students through a practical exercise and finally participants were presented with a practical exercise they had to complete in small groups and later present to the rest of the cohort (Figure 1E). This curriculum design, with real-world case examples and practical sessions, has received excellent feedback from participants and allowed a comparably deep dive in the short time frame of the course. The online version of the course adapted the framework by removing the breakout sessions and including online questionnaires for participants to practice individually. Importantly, all course content was collected on an open platform and remains available to everyone under an open source CC-BY-4.0 license.

In more detail, the curriculum covered the following content: Day 1 was data-focused and began by introducing foundational concepts on the processing of chemical data sets for them to be suitable for AI/ML approaches. This included content around the sources of public data, data cleaning, featurization, chemical space, and comparing data using dimensionality reduction techniques. Day 2 progressed to fundamental ML concepts such as supervised versus unsupervised methods, specific modeling techniques in each paradigm, model performance metrics, and learning to read ML publications. Day 3 covered a broader scope of AI tools that had strong applicability to participants’ current research (including the Ersilia Model Hub) and then required participants to conduct a virtual screening exercise using relevant models in the Hub. Lastly, Day 4 introduced examples of the current state-of-the-art AI research for drug discovery and then closed with a consolidation period to recap the week’s learnings.

### AI Platform with a Graphical User Interface

To deliver the practical components of the course, we developed two no-code assets: (i) educational Streamlit apps aimed at offering step-by-step practical examples as part of hands-on sessions and group activities and (ii) a Graphical User Interface (GUI) to the Ersilia Model Hub, an open source platform containing over 200 research-ready AI/ML models for drug discovery.

The interactive apps were adapted to the participants’ background and needs and covered basic concepts of ML (unsupervised and supervised), chemical similarity search, model training and cross-validation, and sampling the chemical space. Streamlit apps are thoroughly documented, remain available in Streamlit Cloud but are not intended for research use outside the specific context of the exercise (Figure 2A).

**Figure 2:**
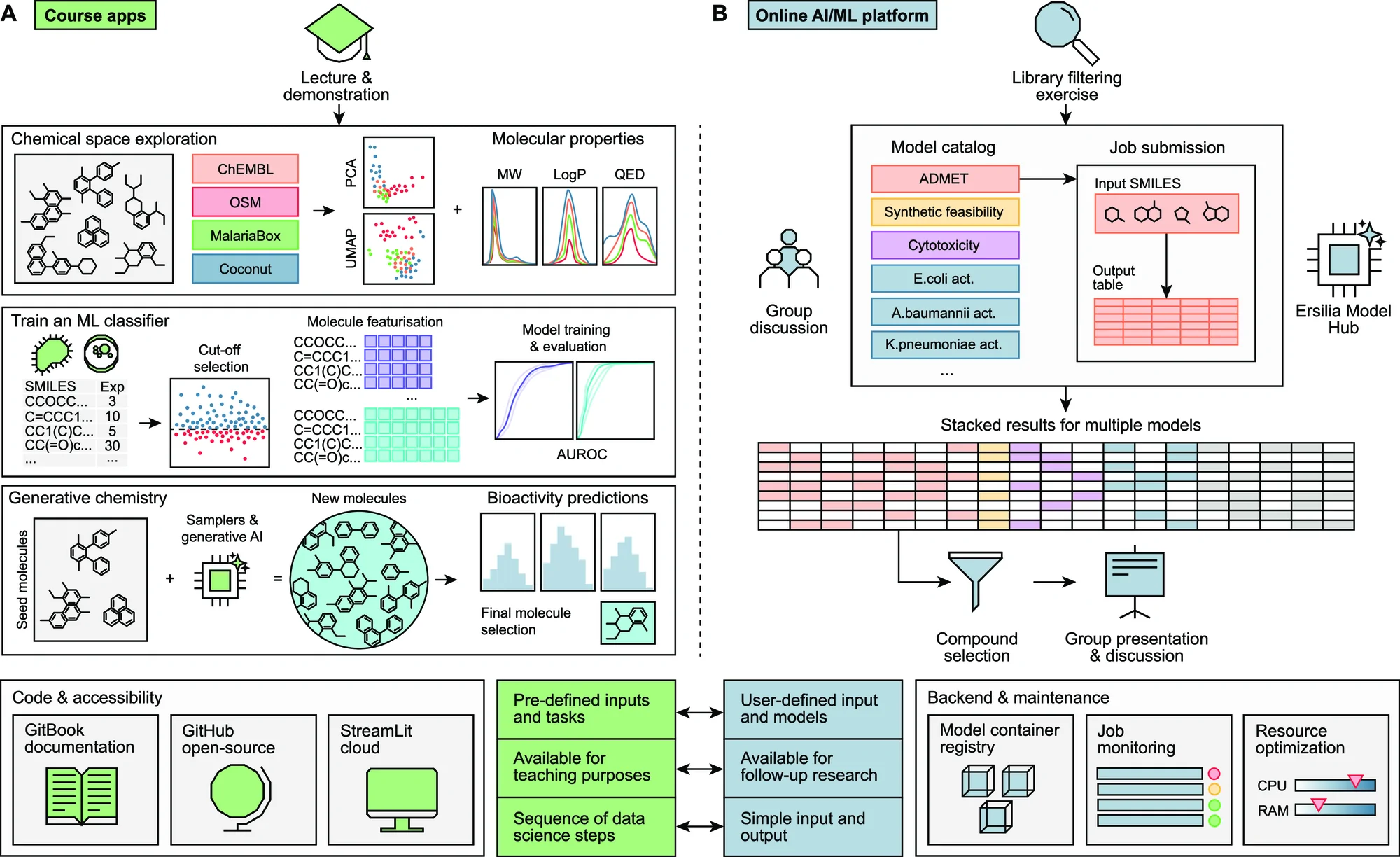
Scheme of the computational platforms developed for the workshops. (A) Course apps were specifically developed to accompany lectures. These apps consist of multistep workflows with predefined inputs and tasks and are available for teaching purposes in the open source domain. (B) An AI/ML platform for model serving was made available to the participants to enable real-world application of AI/ML models. The platform exposes models from the Ersilia Model Hub, deployed as Docker containers with a maintainer-facing interface for job monitoring and resource optimization. The platform remains available to participants for their own follow-up research.

On the other hand, access to research-ready AI/ML models was facilitated by the Ersilia Model Hub,^10^ a collection of bioactivity prediction, molecule representation and generative models focused on infectious disease drug discovery. The Ersilia Model Hub is available through a simple command line interface (CLI) for UNIX systems; but, as discussed above, this posed a major barrier to many medicinal chemists and resulted in low engagement and adoption beyond the course. For this reason, we developed a GUI that accesses a selection of AI/ML models served in the cloud and exposes them in a simple interface that takes as input a list of molecules in SMILES format and produces, as output, tabular files for easy exploration as spreadsheets (Figure 2B). The Ersilia GUI was demonstrated on examples related to antibiotic activity and ADMET prediction (Table 1).

**Table 1:** Representative Set of Models from the Ersilia Model Hub Available to Course Participants Through the GUI Platform

identifier	short description	relevance to workshop
eos3804	Inhibition of *Acinetobacter baumannii* growth.^14^	Primary activity end point for group exercises focused on Gram-negative bacteria.
eos4e40	Broad spectrum antibiotic activity, trained on *E. coli* data.^15^	Discuss whether model organism predictions (*E. coli*) can be extrapolated to other Gram-negative bacteria.
eos18ie	Antibiotic activity prediction against *Staphylococcus aureus*.^16^	Antibacterial activity end point of lesser relevance (i.e., Gram-positive pathogen).
eos42ez	Human cytotoxicity end points.^16^	Explore correlation between cytotoxicity in human cells and antibacterial activity.
eos7m30	ADMET-AI model predicting a compendium of 40+ properties and liabilities.^17^	Array of ADMET predictions to prompt discussion on which ones are most relevant in early screening stages.
eos43at	hERG blockade prediction based on public data.^18^	Frequent cardiotoxicity liability associated with a typically costly assay.
eos9ei3	Simple synthetic feasibility score.^19^	One of several synthetic accessibility scores, to critically evaluate hits for their ease of synthesis.
eos9yui	Natural product likeness score based on characteristic fingerprints.^20^	Distinguish between natural product-like compounds, and establish debate as to their suitability as hits.
eos2db3	2D projections (PCA, UMAP and tSNE) onto a ChemDiv diversity library.	Visual asset to aid group presentations and comment on chemical diversity, clustering, and class (active/inactive) separation.

To provide continued support for the Ersilia GUI, a “maintainer view” was developed to incorporate more models into the deployment cycle, optimize cloud computing usage, visualize logs for troubleshooting, and enable caching functionality. Course participants were encouraged to request models and test the tool with their compounds of interest. We have found this intuitive tool boosts participant engagement and is one of the main outcomes of this training series. Moreover, it is made freely available to any interested researcher, with a cap of 1000 molecules per run. Medicinal chemists interested in using the Ersilia Model Hub through the GUI or locally for more data-intensive procedures are encouraged to contact the authors.

### Participant Feedback

Feedback was collected after each course and used to refine the design of subsequent offerings. Across the in-person workshops in Ghana (September 2024) and Kenya (July 2025), as well as the online webinar (September 2025), participants consistently reported strong enthusiasm for the tools and concepts introduced. One participant in the Kenyan workshop described the experience as opening “a wonderful new world,” while a webinar participant noted that the training “moved the concept from a buzzword in literature to a tangible, operable workflow.”

Participants particularly valued the balance between conceptual grounding and practical application. As one attendee reflected, “The balance between theory (ML concepts, model evaluation) and application (predicting activity, visualizing chemical space) was very effective.” Breakout sessions, in which participants worked directly with data sets and AI-enabled workflows, were consistently identified as the most impactful component of the courses. These hands-on exercises helped translate abstract machine learning concepts into practical tools for medicinal chemistry: “The hands-on application made the often-abstract concepts of machine learning incredibly concrete and immediately relevant.” Participants also noted the value of the collaborative format of the breakout sessions, with one Ghana workshop attendee commenting that “the breakout sessions gave us the opportunity to learn from our peers,” while suggesting that additional time for these exercises would be valuable.

For the in-person workshops, networking opportunities emerged as another major benefit. Participants appreciated the opportunity to connect with peers working on related problems and expressed interest in maintaining these connections through informal channels, such as messaging groups, to continue exchanging knowledge and troubleshooting challenges.

Quantitative feedback reinforced these qualitative impressions. For the Ghana and Kenya workshops, all respondents rated the overall benefit of the course as either 4 (10–22%) or 5 (78–90%) out of 5, and all respondents indicated that they would recommend the course to colleagues. Webinar participants reported similarly positive perceptions, with 75% rating the course as 5 out of 5, 22% as 4 out of 5, and only 3% as 3 out of 5. Nearly all respondents (99.9%) stated that they would recommend the course (Figure 3A).

**Figure 3:**
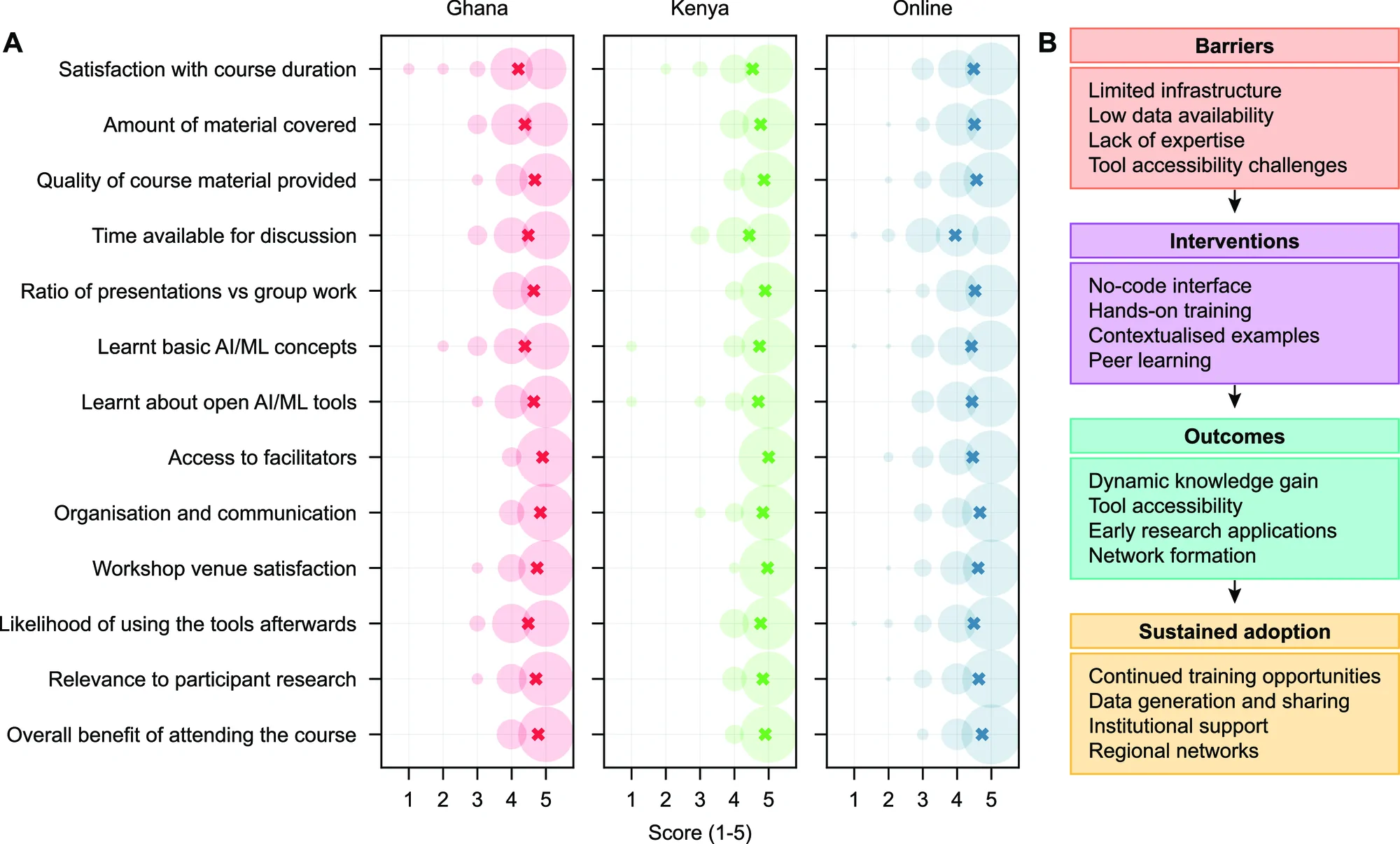
Satisfaction levels and theory of change. (A) Survey scores (1: lowest, 5: highest) resulting from the three workshops (two in-person, one online). The radius of the bubble’s scales with the proportion of votes. The cross indicates the average score. (B) Overall capacity strengthening logic, starting from the barriers and ending with sustained adoption of the learnings and tools.

Several recurring requests also emerged from participants’ feedback. Many respondents expressed a desire for longer courses that would allow more time for hands-on exercises and deeper exploration of specific tools and workflows. Participants also highlighted the importance of continued support following the training, particularly for implementing AI-enabled approaches within their own research projects. Finally, some attendees suggested parallel tracks or advanced modules that would allow participants with different levels of prior experience to progress at an appropriate pace.

Overall, participant feedback indicates that hands-on engagement with AI workflows, opportunities for peer interaction, and sustained post-training support are critical elements for effective capacity building in AI-enabled drug discovery.

## Implications for the Medicinal Chemistry Community

The primary goal of our capacity strengthening initiative was to equip lab-based scientists with the skills to apply AI/ML approaches within their own research contexts. The appetite for data science and AI/ML training in Africa, reflected in the overwhelming number of applications for our workshops, particularly among emerging researchers, highlights the opportunities and urgency in this space. Motivated by the response to our course offerings, we have shared our experience in this perspective to inform the broader scientific community and encourage medicinal chemists to review and adapt the assets presented here to their context.

Based on the course feedback, we believe this training initiative is a valuable starting point to addressing a key gap in capacity and expertise toward sustainable adoption of AI/ML for drug discovery in Africa.^11^ In particular, the tools developed for these training activities, including the GUI to the Ersilia Model Hub, lower barriers of entry and enable researchers to begin incorporating AI/ML predictions in their medicinal chemistry initiatives. By embedding these resources within the open-source Ersilia ecosystem and supporting them through a cloud-based backend, we aim to provide a durable, globally accessible platform that can be adopted, adapted, and expanded by the medicinal chemistry community (Figure 3B).

Drug discovery capacity on the continent is still growing,^12^ with limited data generation capabilities and infrastructure gaps being a major constraint. A combination of training, concerted efforts in data sharing and the strengthening of research infrastructure is required to address this and enable the transition toward AI/ML-driven drug discovery in Africa.

### Looking Ahead

The initial success of this training series will only be fully accomplished if subsequent initiatives and capacity development opportunities arise for both current and future participants. Given the appreciation for personal contact (both facilitator-participant and peer-to-peer) through in-person workshop sessions and networking opportunities, as well as the expressed desire for longer workshops, future program designs could move beyond short-format workshops toward extended, cohort-based models, spanning several months and supported by regular touchpoints. Such programs would enable participants to develop a deeper understanding of AI/ML tools and facilitate their effective, barrier-free implementation within their home institutions. We envision these efforts being strengthened through collaboration with existing networks, such as the Grand Challenges African Drug Discovery Accelerator (https://gcadda.org) initiative, to ensure continuity, mentorship, and regional integration. Furthermore, this collaborative approach with existing networks will facilitate the wet-lab/dry-lab cycle, enabling the collection and publication of meaningful experimental data for the African context.

At the same time, the diversity of participant backgrounds, in terms of both previous expertise and career stage, points to the need for more personalized training pathways. While the application-focused, no-code approach is beneficial to all participants, a subset of trainees with computational backgrounds could obtain more advanced, programming-oriented training. Addressing this need represents an important opportunity to cultivate local expertise capable of developing and adapting AI/ML models beyond implementation of existing tools alone. This could be achieved either by extending the current workshop format (e.g., with an additional advanced module) or by offering dedicated, standalone courses targeting this audience.

Looking ahead, several complementary avenues for program expansion can be considered, from continuing the in-person, short-term workshops to meet the growing demand for training, to offering longer-term, tailored programs. A version of the latter has been piloted through a bespoke program delivered specifically to H3D chemists. With support from the AI2050 Collaborative Fund at Schmidt Sciences, an international training team composed of facilitators from H3D, Ersilia and Massachusetts Institute of Technology (MIT) developed a 3-day training to consolidate institutional integration of AI/ML platforms in the H3D Centre.^13^ Finally, an incubator-style model could provide structured follow-on support to workshop alumni, guiding them from initial exposure to the successful implementation of AI/ML tools within their research environments.

## Data Availability

The GUI used in the courses can be accessed at: https://hub.ersilia.io. Course materials, including data sets, slide decks, and related code, are centralized via the AI2050 “AI for drug discovery” workshops documentation at https://ersilia.gitbook.io/ersilia-workshops/ai2050-program, including the links to webinar recordings. Code to prepare the figure plots is publicly available at https://github.com/ersilia-os/ai2050-workshop-outcome.
